# Submaximal exercise cardiac output is increased by 4 weeks of sprint interval training in young healthy males with low initial *Q̇*-*V̇*O_2_: Importance of cardiac response phenotype

**DOI:** 10.1371/journal.pone.0195458

**Published:** 2019-01-23

**Authors:** Robert F. Bentley, Joshua H. Jones, Daniel M. Hirai, Joel T. Zelt, Matthew D. Giles, James P. Raleigh, Joe Quadrilatero, Brendon J. Gurd, J. Alberto Neder, Michael E. Tschakovsky

**Affiliations:** 1 School of Kinesiology and Health Studies, Human Vascular Control Laboratory, Queen’s University, Kingston, ON, Canada; 2 Department of Medicine, Division of Respirology, Laboratory of Clinical Exercise Physiology, Queen’s University, Kingston, ON, Canada; 3 School of Kinesiology and Health Studies, Queen’s Muscle Physiology Laboratory, Queen’s University, Kingston, ON, Canada; 4 Department of Kinesiology, Muscle Biology and Cell Death Laboratory, University of Waterloo, Waterloo, ON, Canada; Universita degli Studi di Roma 'Foro Italico', ITALY

## Abstract

Cardiovascular adaptations to exercise, particularly at the individual level, remain poorly understood. Previous group level research suggests the relationship between cardiac output and oxygen consumption ($\dot{Q}$-$\dot{V}O_{2}$) is unaffected by training as submaximal $\dot{Q}$ is unchanged. We recently identified substantial inter-individual variation in the exercise $\dot{Q}$-$\dot{V}O_{2}$ relationship that was correlated to stroke volume (SV) as opposed to arterial oxygen content. Therefore we explored the effects of sprint interval training (SIT) on modulating $\dot{Q}$-$\dot{V}O_{2}$ given an individual’s specific $\dot{Q}$-$\dot{V}O_{2}$ relationship. 22 (21±2 yrs) healthy, recreationally active males participated in a 4-week SIT (8, 20 second sprints; 4x/week, 170% of the work rate at $\dot{V}O_{2}$ peak) study with progressive exercise tests (PET) until exhaustion. Cardiac output ($\dot{Q}$ L/min; inert gas rebreathe, Finometer Modelflow™), oxygen consumption ($\dot{V}O_{2}$ L/min; breath-by-breath pulmonary gas exchange), quadriceps oxygenation (near infrared spectroscopy) and exercise tolerance (6–20; Borg Scale RPE) were measured throughout PET both before and after training. Data are mean Δ from bsl±SD. Higher $\dot{Q}$ ($H\dot{Q}$) and lower $\dot{Q}$ ($L\dot{Q}$) responders were identified *post hoc* (n = 8/group). SIT increased the $\dot{Q}$-$\dot{V}O_{2}$ post-training in $L\dot{Q}$ (3.8±0.2 vs. 4.7±0.2; P = 0.02) while $H\dot{Q}$ was unaffected (5.8±0.1 vs. 5.3±0.6; P = 0.5). $\Delta \dot{Q}$ was elevated beyond 80 watts in $L\dot{Q}$ due to a greater increase in SV (all P<0.04). Peak $\dot{V}O_{2}$ (ml/kg/min) was increased in $L\dot{Q}$ (39.7±6.7 vs. 44.5±7.3; P = 0.015) and $H\dot{Q}$ (47.2±4.4 vs. 52.4±6.0; P = 0.009) following SIT, with $H\dot{Q}$ having a greater peak $\dot{V}O_{2}$ both pre (P = 0.02) and post (P = 0.03) training. Quadriceps muscle oxygenation and RPE were not different between groups (all P>0.1). In contrast to $H\dot{Q}$, $L\dot{Q}$ responders are capable of improving submaximal $\dot{Q}$-$\dot{V}O_{2}$ in response to SIT via increased SV. However, the increased submaximal exercise $\dot{Q}$ does not benefit exercising muscle oxygenation.

## Introduction

Systemic oxygen delivery, the product of arterial oxygen content (C_a_O_2_) and cardiac output ($\dot{Q}$), increases in proportion to exercising muscle oxygen consumption ($\dot{V}O_{2}$) and is closely coupled to the increase in exercising muscle blood flow [1, 2]. This matching is a key determinant of the ability to sustain exercise [3]. Consistent with this, the perceived psychophysiological stress of exercise, as quantified by the Borg scale of ratings of perceived exertion (RPE) exercise [4, 5], is sensitive to exercising muscle oxygen delivery and oxygenation, with hypoxia increasing [6] and hyperoxia decreasing [7] RPE for a given work rate. Understanding factors that improve or compromise this matching is therefore of substantial importance for addressing issues of exercise tolerance. At present, the “typical” slope of the $\dot{Q}$ response to an increase in oxygen demand across individuals is ~5–7 L/min of $\dot{Q}$ per 1 L/min of $\dot{V}O_{2}$ [8–10]. Within this expected range during exercise, inter-individual differences are thought to reflect [hemoglobin]-dependent differences in C_a_O_2_ between individuals [8–10] as hypoxia increases the $\dot{Q}$-$\dot{V}O_{2}$ slope [10].

However, we recently demonstrated that healthy young recreationally active males present with $\dot{Q}$-$\dot{V}O_{2}$ ranging from 3.3 to 7.0 L/min of $\dot{Q}$ per 1 L/min of $\dot{V}O_{2}$ [11], which aligns with previous work from the 1960s [12, 13] and 1980s [14]. Importantly, we demonstrated that $\dot{Q}$-$\dot{V}O_{2}$ was not related to C_a_O_2_ [11] whereas stroke volume (SV) and total vascular conductance were strongly correlated with $\dot{Q}$ across a range of submaximal exercise intensities. Functionally, having a lower $\dot{Q}$-$\dot{V}O_{2}$ was associated with a lower peak $\dot{V}O_{2}$ [11]. These phenotype response differences are entirely consistent with the demonstrated cardiac β-2 receptor polymorphism findings in which individuals homozygous for arginine at codon 16 present with lower submaximal exercise $\dot{Q}$ [15].

Endurance exercise is a conventional training method that consistently produces increases in peak $\dot{V}O_{2}$ and peak $\dot{Q}$ [16–20]. However, $\dot{Q}$-$\dot{V}O_{2}$ is thought not to change [16, 18], as submaximal $\dot{Q}$ for a given $\dot{V}O_{2}$ does not change, or is reduced [16–19, 21]. Sprint interval training (SIT) is a different exercise approach that results in similar if not better cardiovascular improvements as typical endurance training in a fraction of the exercise time [22, 23]. The Tabata protocol [24] is a common training modality that involves supramaximal 20 second sprints on a cycle ergometer at an intensity equivalent to 170% of the work rate at peak $\dot{V}O_{2}$ separated by a brief, 10 second active recovery period.

To date, only one study has explored the impact of SIT on submaximal exercise $\dot{Q}$. In obese sedentary women, where the group was examined as a whole, there was no change in submaximal $\dot{Q}$, as increases in SV were offset by reductions in heart rate (HR). There was however an improvement in peak $\dot{V}O_{2}$ [21]. Importantly, neither this nor any other studies have considered the influence of an individual’s $\dot{Q}$-$\dot{V}O_{2}$ relationship on submaximal $\dot{Q}$ response to exercise training.

Therefore, the purpose of this study was to determine the effects of individual differences in $\dot{Q}$-$\dot{V}O_{2}$ on the cardiovascular adaptation to SIT. A secondary objective was to explore how potential SIT-mediated changes in $\dot{Q}$ affect submaximal skeletal muscle oxygenation and RPE. We hypothesized that individuals with a relatively lower $\dot{Q}$-$\dot{V}O_{2}$ would present with a greater SV post-training, presumably due to central adaptions, thus transforming them into a higher cardiac responder during subsequent submaximal exercise. Individuals with a relatively higher $\dot{Q}$-$\dot{V}O_{2}$ would not be centrally affected by SIT and their $\dot{Q}$-$\dot{V}O_{2}$ would remain unchanged during subsequent exercise post-training. As a result of the elevated $\dot{Q}$ in lower cardiac responders post-training, exercising skeletal muscle oxygenation would be increased while local RPE would be reduced. This information will aid in identifying functional training programs tailored to individuals, that improve oxygen delivery during exercise.

## Methods

### Participants

31 participants were recruited to participate in this study. 22 healthy, recreationally active (< 3 hours/week of structured exercise) male participants (21 ± 2 yrs) with no history of smoking, cardiovascular disease or hypertension completed the study. 8 individuals dropped out citing time constraints (n = 4), lack of desire to continue (n = 2), negative side effects associated with training (n = 1; headaches), not feeling well enough to complete the post-training progressive exercise test (n = 1). 1 participant was removed *post hoc* due to data quality. Participants for this study for all intent and purposes were a homogeneous male subset from our population. This study was approved by the Health Sciences Research Ethics Board at Queen's University according to the terms of the Declaration of Helsinki. Procedures were in accordance with institutional guidelines. Each subject provided signed consent after receiving complete verbal and written descriptions of the experimental protocol and potential risks.

### Experimental design

This was a 4 week SIT study. Participants completed a venous blood sample and a progressive exercise test to exhaustion both before and after SIT. Post-testing venous blood sample was taken 72 hours following training cessation while a progressive exercise test to exhaustion was completed after 96 hours. Progressive exercise tests were completed at the same time of day within a participant and exercise was avoided for 24 hours prior. To the best of our ability SIT sessions were performed at the same time of day within a participant. A single progressive exercise test was completed due to the strong reproducibly in the assessment of $\dot{Q}$ and $\dot{V}O_{2}$ during prior bouts of submaximal cycling (combined within and between day coefficient of variation of 2.2% and 2.8% respectively (pilot work n = 4)). Given the low level of variability, modified Monte Carlo simulations determined that multiple repeats were not required to state with confidence that a particular individual had a different cardiac response from someone else.

#### Progressive exercise test protocol (Fig 1; Panel A)

A progressive exercise test to exhaustion was completed before and after training. The day after the venous blood sample, participants arrived at Kingston General Hospital for a progressive exercise test to exhaustion. Each participant completed progressive cycling exercise on an electronically braked cycle ergometer (VIAsprint 150P; Ergoline, Bitz, Germany). Seat height was adjusted for each participant to a level deemed comfortable by each participant and recorded for post-testing. Participants rested on the bike for 5 minutes. Exercise started at 40 watts (W) (at a self-selected rpm during pre-testing which was recorded for post-testing) and increased by 40 W every 4 minutes until 160 W. Following 160 W, exercise intensity increase by 25 W every minute until volitional exhaustion. During exercise systemic blood pressure, $\dot{Q}$, and pulmonary gas exchange were measured.

**Fig 1:**
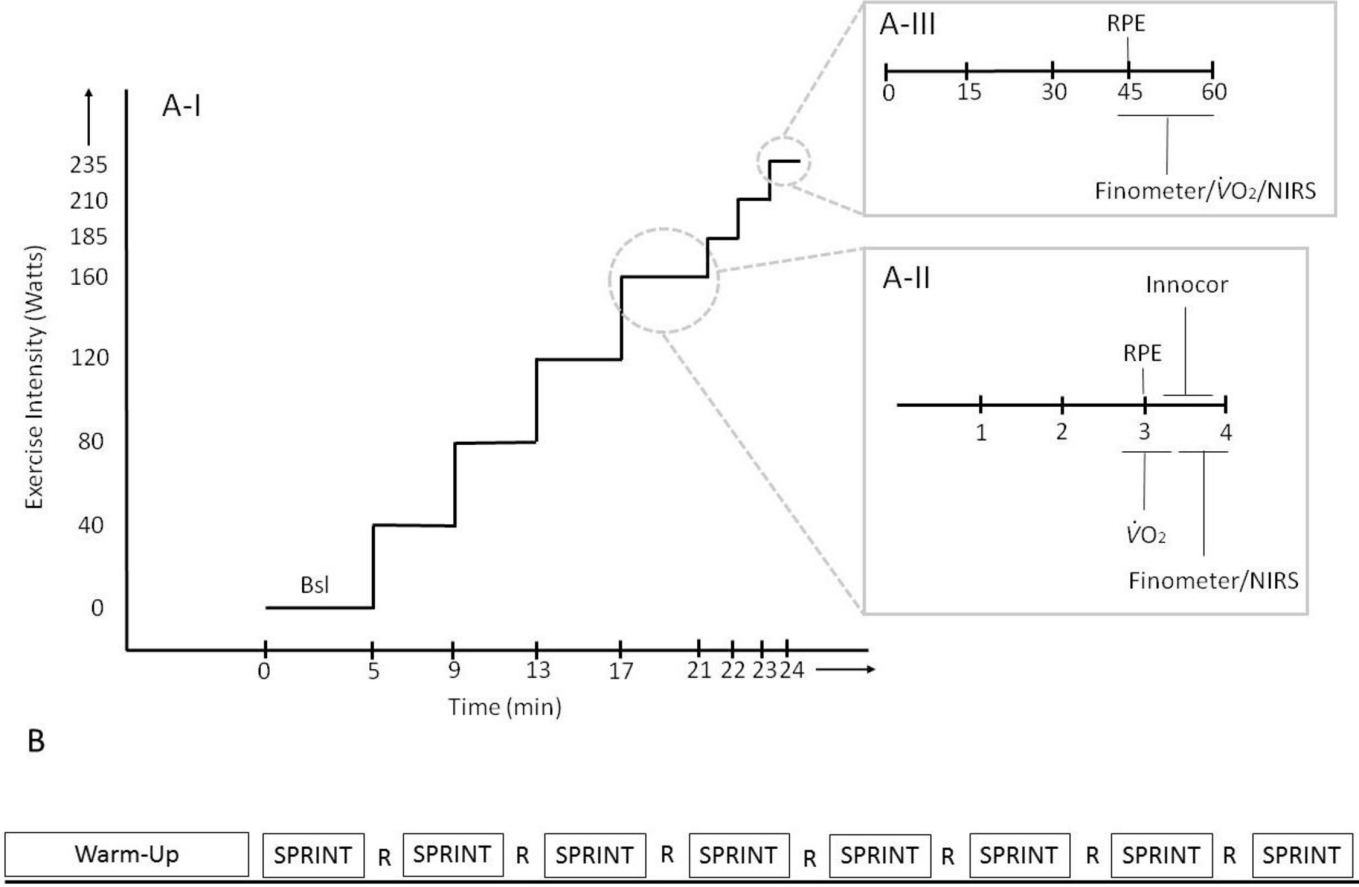
Experimental protocols. *Panel A-I*: Progressive exercise test beginning with rest and increasing by 40 W every 4 minutes until 160 W. Beyond 160 W, exercise increased by 25 W every minute until volitional exhaustion. *Panel A-II*: Timing of measurements (in minutes) during exercise up to 160 W. *Panel A-III*: Timing of measurements (in seconds) during exercise beyond 160 W. *Panel B*: Sprint interval training. 5 minute warm up is followed by successive 8, 20 second intervals at an intensity associated with the work rate at 170% of peak $\dot{V}O_{2}$ at 80 rpm. These were separated by 10 seconds of rest (loadless cycling—active recovery) at a self-selected rpm, for a total of 4 minutes of exercise. RPE; ratings of perceived exertion, $\dot{V}O_{2}$; oxygen consumption, NIRS; near infrared spectroscopy, R; active recovery.

#### Sprint interval training protocol (Fig 1; Panel B)

Supervised training was performed 4 times per week for 4 weeks, for a total of 16 SIT sessions. Participants completed 8, 20 second intervals at an intensity associated with the work rate at 170% of peak $\dot{V}O_{2}$ at 80 rpm on a stationary cycle ergometer (Monark, Ergomedic 874E; Vansbro, Sweden). These were separated by 10 seconds of rest (loadless cycling—active recovery) at a self-selected rpm, for a total of 4 minutes of exercise. Each sprint was completed at 80 rpm. Successive sprint repeats resulted in the gradual inability to maintain 80 rpm. In any event, sprinting was completed for the full 20 second interval followed by a rest period. A 5 minute warm-up preceded the sprint intervals.

### Instrumentation and data acquisition

#### Standard anthropometric data

Upon arrival in the laboratory, standard anthropometric data was obtained for each subject. Age, height and weight were measured while a 7-day physical activity recall adapted from Sarkin *et al*. [25] was completed to quantify current exercise habits.

#### Venous blood sample

The day prior to the progressive exercise test, a supine resting blood sample was obtained for each participant using standard venipuncture into a 4.5 ml lithium heparin vacutainer and immediately analyzed for hemoglobin content with a blood gas analyzer (Stat Profile Prime Blood Gas Analyzer, Nova Biomedical, Mississauga, Canada).

#### Cardiac output–inert gas rebreathe

In a 5-breath maneuver, participants breathed a mixture of 3 gases (99.4% oxygen, 0.5% nitrous oxide, and 0.1% sulfur hexafluoride) from a rebreathing bag (Innocor, Innovision, GbH) as described previously [11]. Breathing maneuvers were completed at rest and during exercise at 40 W increments once every 4 minutes up to 160 W. This rebreathing method correlates well with direct Fick measures of $\dot{Q}$ r = 0.94 [26] with a mean bias of +0.34 ± 0.59 L/min [27] and demonstrates strong reproducibility (coefficient of variation 4.3%) during submaximal exercise in healthy participants [28]. Pilot work (n = 4) demonstrated that our $\dot{Q}$ variability (combined assessment of both within and between day) was 2.2% for exercise intensities up to 200 W with a typical error of 0.36 L/min.

#### Cardiac output–finger photoplethysmography

A finger photoplethysmograph was placed on the middle finger of the left hand to measure mean arterial blood pressure throughout exercise. Both arms were supported by clip-on Bontrager aerobars (Trek, Waterloo, WI) and therefore the hand remained in the same position relative to heart level at all times. The aerobars also allowed for the hands to remain relaxed throughout exercise. In addition to blood pressure, this device provides estimates of SV and thereby computed $\dot{Q}$ and total peripheral resistance (TPR) via the ModelFlow^TM^ (Finapres Medical Systems, The Netherlands).

#### Quadriceps muscle oxygenation status

Quadriceps oxygenation of the right *vastus lateralis* was measured by frequency-domain multidistance near-infrared spectroscopy (FDMD NIRS, Imagent; ISS, Champaign, IL). Principles of operation and algorithms have been described in detail previously [29] and positioning was determined as described previously [11]. FDMD NIRS provides measurements of the absolute concentrations of oxygenated and deoxygenated hemoglobin/myoglobin (oxy-[Hb/Mb] and deoxy-[Hb/Mb], respectively). Measures of total heme concentration (total [Hb/Mb] = oxy-[Hb/Mb] + deoxy-[Hb/Mb]) and tissue oxygen saturation (StO_2_ = oxy-[Hb/Mb]/total [Hb/Mb], %) were also calculated. Oxygenation status of the muscle was recorded continuously throughout exercise.

#### Oxygen consumption and heart rate

Pulmonary gas exchange was measured breath-by-breath using a calibrated, computer-based system (Vmax Encore 229, CareFusion, Yorba Linda, CA) as discussed previously [11]. Pulmonary oxygen uptake ($\dot{V}O_{2}$) was measured continuously. Participants were outfitted with a 4 lead electrocardiogram for the measurements of HR throughout exercise.

#### Ratings of perceived exertion

Three minutes into rest and during steady state exercise at each intensity up to 160 W, ratings of whole body perceived exertion (RPE_WB_) and peripheral perceived exertion of the legs (RPE_L_) were obtained in succession on the Borg 6–20 scale. Prior to exercise participants were oriented to the scale and read a script taken from [30] to ensure correct understanding of exertional assessment. Beyond 160 W, RPE was obtained during the last 15 seconds of an exercise intensity.

### Data analysis

#### Venous effluent–hemoglobin concentration

The resting venous blood sample was analyzed with a blood gas analyzer (Stat Profile Prime Blood Gas Analyzer, Nova Biomedical, Mississauga, Canada) for hemoglobin concentration.

#### Oxygen consumption

Oxygen consumption was obtained on a breath-by-breath basis. At rest and up to 160 W, a 30 second average was computed within the last 1 minute and 30 seconds of an exercise intensity. Beyond 160 W a 15 second average was computed during the last 15 seconds of a completed exercise intensity.

#### Cardiac output–inert gas rebreathe

Pulmonary blood flow (cardiac output; $\dot{Q}$) was measured during the last ~30 seconds of rest and each completed exercise intensity up to 160 W. As discussed previously [11], beyond 160 W inert gas rebreathe was not completed to assess $\dot{Q}$ and as such, the following method was used to obtain measures of $\dot{Q}$ during exercise at higher intensities.

#### Cardiac output–finger photoplethysmography

Finometer measures of arterial blood pressure and estimates of $\dot{Q}$ were obtained on a beat-by-beat basis. $\dot{Q}$ measures were time aligned with Innocor during rest and each measurement up to 160 W. Beyond 160 W, we employed a correction factor to our Finometer estimates of $\dot{Q}$ to provide corrected measures of $\dot{Q}$ at higher exercise intensities similar to Tam *et al*. [31]. Our method of correction consisted of a linear regression of Finometer estimates of $\dot{Q}$ and Innocor measures of $\dot{Q}$ up to 160 W. Once an individual’s relationship was established, given an estimated Finometer measure of $\dot{Q}$, an Innocor corrected Finometer $\dot{Q}$ measure could be computed for work rates above 160 W. Within-subject linear regressions between Innocor measures of $\dot{Q}$ and Finometer estimates of $\dot{Q}$ were excellent (r^2^ = 0.96 ± 0.03).

#### Heart rate and arterial blood pressure and skeletal muscle oxygenation

HR, arterial blood pressure and skeletal muscle oxygenation were computed during the same time period as $\dot{Q}$.

#### Statistical analysis

Statistical analysis was completed on pre-identified cardiac response groups [11]. To identify an individual’s cardiac response, a linear regression was completed for each participant’s $\Delta \dot{Q}$ vs. $\Delta \dot{V}O_{2}$ data from 40 W to 185 W. This exercise intensity range was used because it ensured that all participant fits were based on the same exercise intensity increments, as with increasing exercise intensity there was participant attrition beyond 185 W. Individual $\dot{Q}$-$\dot{V}O_{2}$ slope responses were ranked, and participants with the 8 lowest and 8 highest slopes were grouped *post hoc* as lower and higher cardiac responders respectively. All subsequent analysis was completed on these two groups, unless otherwise noted.

Following *post hoc* identification of lower and higher cardiac response groups, $\Delta \dot{Q}$ was plotted against $\Delta \dot{V}O_{2}$ at each exercise intensity for each group. A linear regression was completed for each group’s data and slope analysis of these regressions was completed to determine if there was a significant difference both between group and with training.

A two way repeated measures mixed model ANOVA was used to compare lower vs. higher cardiac responders at submaximal exercise intensities (40 W to 185 W) before and after training for all cardiovascular variables, skeletal muscle saturation, and RPE. A one way repeated measures mixed model ANOVA was used to compare lower vs. higher cardiac responders before and after training for all baseline cardiovascular variables, and peak responses.

Significance was set at P < 0.05. For all analyses, post measures were matched to pre measures such that the same participant completed each exercise intensity both before and after training. Only significant F-statistics within the repeated measures ANOVA were further assessed using Bonferroni corrected *post hoc* tests. All assumptions of normality and homogeneity of variance in the repeated measures ANOVA were met. In some instances the assumption of sphericity was not met, in which case a Greenhouse-Geisser correction was applied when determining F-statistic significance. Statistics were calculated using a combination of SPSS 20 (IBM Software), SigmaPlot 12.0 (Systat Software, Inc.) and GraphPad Prism 6 (GraphPad Software, Inc.). All results presented are mean Δbsl ± SD unless otherwise noted.

During statistical analysis it was identified that one participant had an implausible $\dot{Q}$ at 160 W pre-training as measured by computed systemic venous oxygen content (C_v_O_2_; 0 < C_v_O_2_ < 20 ml O_2_/L). This participant was removed from all analysis and analysis was therefore completed on n = 22. All remaining 264 computed values of systemic C_v_O_2_, stemming from measured $\dot{Q}$, were plausible (C_v_O_2_ ≥ 20 ml O_2_/L), providing confidence in the $\dot{Q}$ measures in the present study (see experimental considerations section).

## Results

### Standard anthropometric data, baseline cardiovascular values and peak responses

Standard anthropometric data is presented in Table 1. There was no difference in any parameter before and after training (all P > 0.5). Baseline and peak cardiovascular parameters are presented in Table 2. Prior to exercise training, HR_BSL_ was elevated in lower cardiac responders compared to higher cardiac responders (P = 0.003). Both peak $\dot{Q}$ and peak $\dot{V}O_{2}$ were greater in higher cardiac responders (both P < 0.02). Following exercise training, total vascular conductance at rest was elevated in higher cardiac responders compared to lower cardiac responders (P = 0.02) and in comparison to their pre-training response (P = 0.02). Both groups had an increase in peak $\dot{V}O_{2}$ post-training (both P < 0.01), with higher cardiac responders having a higher peak $\dot{V}O_{2}$ (P = 0.03). Peak $\dot{Q}$ was elevated in lower cardiac responders following training (P = 0.007), but was still lower than higher cardiac responders (P = 0.04).

**Table 1:** Anthropometric measures.

Variable	Group Pre (n = 22)	Group Post (n = 22)	Lower Pre (n = 8)	Lower Post (n = 8)	Higher Pre (n = 8)	Higher Post (n = 8)
Age (yrs)	20 ± 2	---	21 ± 2	---	21 ± 2	---
Height (cm)	182 ± 7	---	181 ± 8	---	181 ± 6	---
Weight (kg)	79.6 ± 10.9	79.4 ± 11.2	78.3 ± 12.9	78.3 ± 13.4	82.6 ± 6.8	82.7 ± 7.7
BMI	23.9 ± 2.5	23.8 ± 2.6	23.7 ± 2.9	23.7 ± 2.9	25.1 ± 1.2	25.1 ± 1.5
7 day PAR score (METS/wk)	247 ± 15	---	244 ± 14	---	250 ± 16	---

Values are mean ± SD. BMI; body mass index, PAR; physical activity recall, METS; metabolic equivalents. NS, P > 0.5 for all comparisons between and within lower and higher cardiac responders.

**Table 2:** Baseline cardiovascular variables and peak responses.

Variable	Group Pre (n = 22)	Group Post (n = 22)	Lower Pre (n = 8)	Lower Post (n = 8)	Higher Pre (n = 8)	Higher Post (n = 8)
MAP_BSL_ (mmHg)	92 ± 10	89 ± 9	97 ± 10	94 ± 9	91 ± 9	87 ± 9
HR_BSL_ (bpm)	85 ± 12	84 ± 13	94 ± 12	90 ± 15	75 ± 8^	81 ± 9
SV_BSL_ (ml/bt)	76 ± 16	79 ± 18	74 ± 23	72 ± 23	83 ± 12	87 ± 17
${\dot{Q}}_{\mathrm{BSL}}$ (L/min)	6.3 ± 1.2	6.5 ± 1.0	6.8 ± 1.7	6.3 ± 1.3	6.2 ± 1.0	6.9 ± 0.9
TVC_BSL_ (L/min/100mmHg)	7.0 ± 1.2	7.3 ± 1.1	7.0 ± 1.5	6.6 ± 1.2	6.8 ± 1.0	8.0 ± 0.9^*
StO_2BSL_ (%)	71 ± 4	73 ± 4	71 ± 4	71 ± 6	70 ± 5	73 ± 2
C_a_O_2_ (mlO_2_/L)	206 ± 11	207 ± 7	207 ± 6	207 ± 4	205 ± 14	208 ± 9
$\dot{V}O_{2}\mathrm{pk}$ (ml/kg/min)	44.0 ± 6.3	48.1 ± 7.7*	39.7 ± 6.7	44.5 ± 7.3*	47.2 ± 4.4^	52.4 ± 6.0^*
$\dot{Q}\mathrm{pk}$ (L/min)	21.2 ± 3.5	21.9 ± 3.3	18.2 ± 2.6	20.8 ± 2.9*	24.3 ± 2.6^	24.3 ± 3.3^

Values are mean ± SD. MAP; mean arterial pressure, HR; heart rate, SV; stroke volume, $\dot{Q}$; cardiac output, TVC; total vascular conductance, StO_2_; exercising skeletal muscle saturation, C_a_O_2_; arterial oxygen content, $\dot{V}O_{2}\mathrm{pk}$; peak rate of oxygen consumption, $\dot{Q}\mathrm{pk}$; peak cardiac output.

* denotes statistical significant difference between pre vs. post-training within a cardiac response group (P < 0.05).

^ denotes statistical significant difference between cardiac response groups at given time period (P < 0.05).

### Sensitivity of cardiac response groups to exercise training

**Fig 2:**
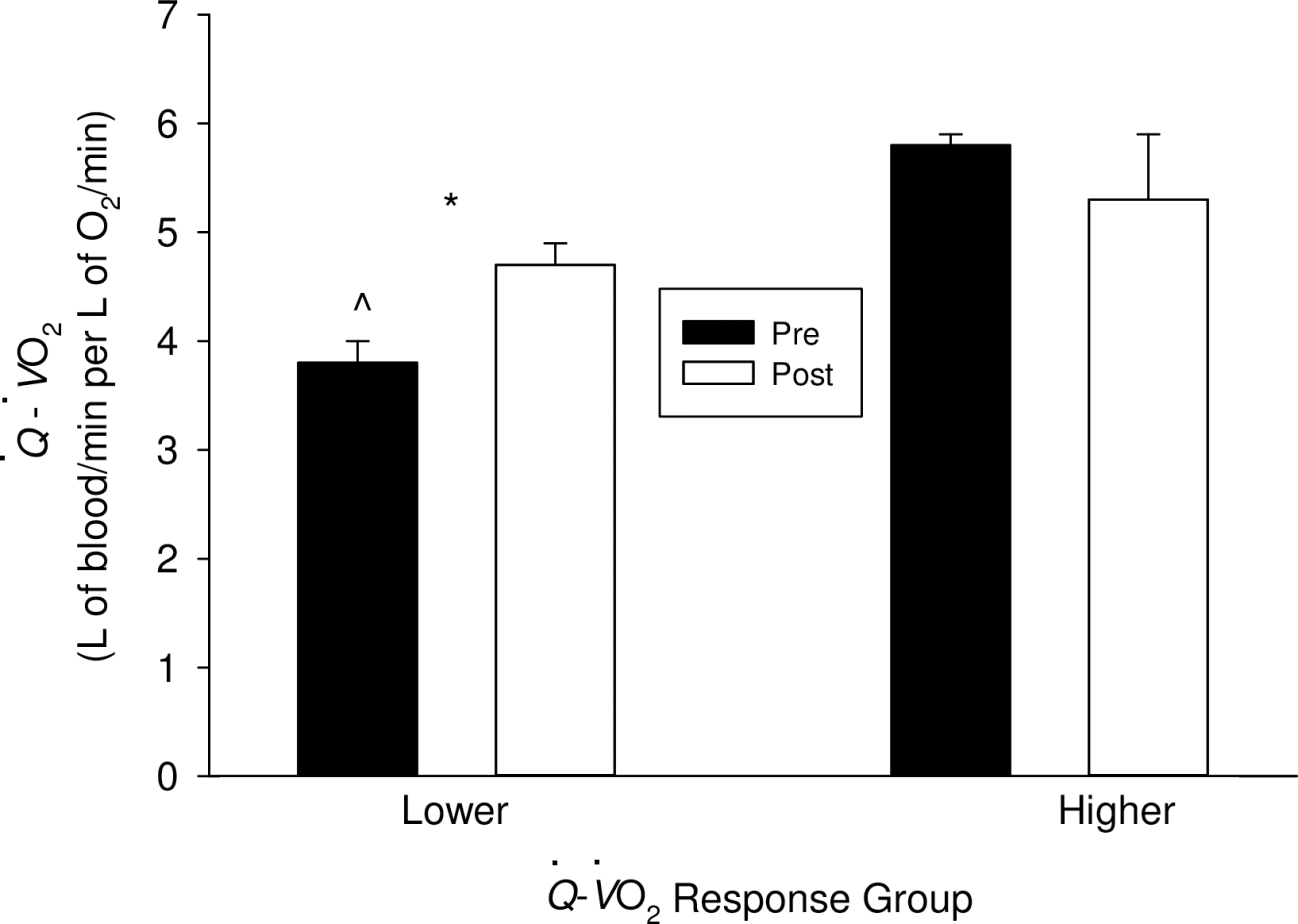
Exercise training and $\dot{Q}$-$\dot{V}O_{2}$. ^ denotes statistically significant difference between lower and higher cardiac response groups within a time period (P < 0.05). * denotes statistically significant difference between pre and post-training within a cardiac response group (P < 0.05).

Prior to exercise training, lower and higher cardiac response groups differed in their $\dot{Q}$-$\dot{V}O_{2}$ (L/min of blood per L/min of O_2_) (P < 0.001). Following exercise training, lower cardiac responders had an increase in their $\dot{Q}$-$\dot{V}O_{2}$ (P = 0.02) while higher cardiac responders’ $\dot{Q}$-$\dot{V}O_{2}$ was unaffected (P = 0.5) (Fig 2). Upon the completion of training, $\dot{Q}$-$\dot{V}O_{2}$ was no longer different between cardiac response groups (P = 0.3).

### Exercise training and the submaximal cardiac response

**Fig 3:**
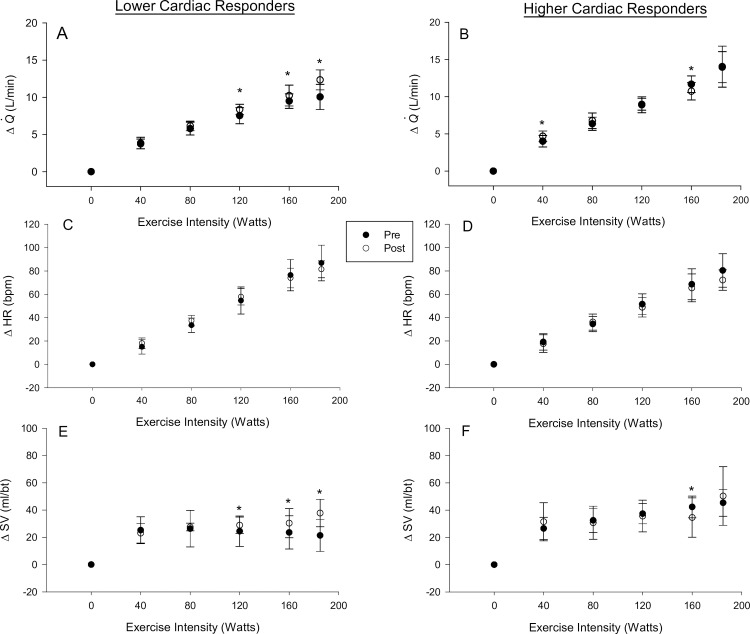
Exercise training and cardiac output constituents. *Panel A-B*: Cardiac output. *Panel C-D*: Heart rate. *Panel E-F*: Stroke volume. Panel *A*, *C*, *E*: Lower cardiac responders. *Panel B*, *D*, *F*: Higher cardiac responders. * denotes statistically significant difference between pre and post-training within a cardiac response group (P < 0.05).

Lower cardiac responders had an increase in $\Delta \dot{Q}$ (L/min) at 120 W (P = 0.01), 160 W (P = 0.04) and 185 W (P = 0.001). This was due to an increase in ΔSV (ml/bt) at 120 W (P = 0.04), 160 W (P = 0.03) and 185W (P = 0.003) as ΔHR was unchanged (F test P = 0.8) (Fig 3). Higher cardiac responders had an increase in $\Delta \dot{Q}$ (L/min) at 40 W (P = 0.046) and a reduction in $\Delta \dot{Q}$ at 160 W (P = 0.017). ΔSV (ml/bt) was reduced at 160 W (P = 0.016) while ΔHR was unchanged (F test P = 0.8) (Fig 3). While $\Delta \dot{Q}$ was reduced in lower cardiac responders compared to higher cardiac responders pre-training at 120 W (P = 0.018), 160 W (P = 0.002) and 185 W (P = 0.001), it was not different post-training (all P > 0.2). Similarly, ΔSV was reduced in low cardiac responders compared to higher cardiac responders pre-training at 120 W (P = 0.03), 160 W (P = 0.006) and 185 W (P = 0.001), but was not different post-training (all P > 0.2). ΔHR was not different between groups (F test P = 0.8). In response to exercise training there was no change in submaximal Δ mean arterial pressure (F test P = 0.2) or submaximal $\dot{V}O_{2}$ (F test P = 0.4) at each exercise intensity.

### Exercise training and skeletal muscle

**Fig 4:**
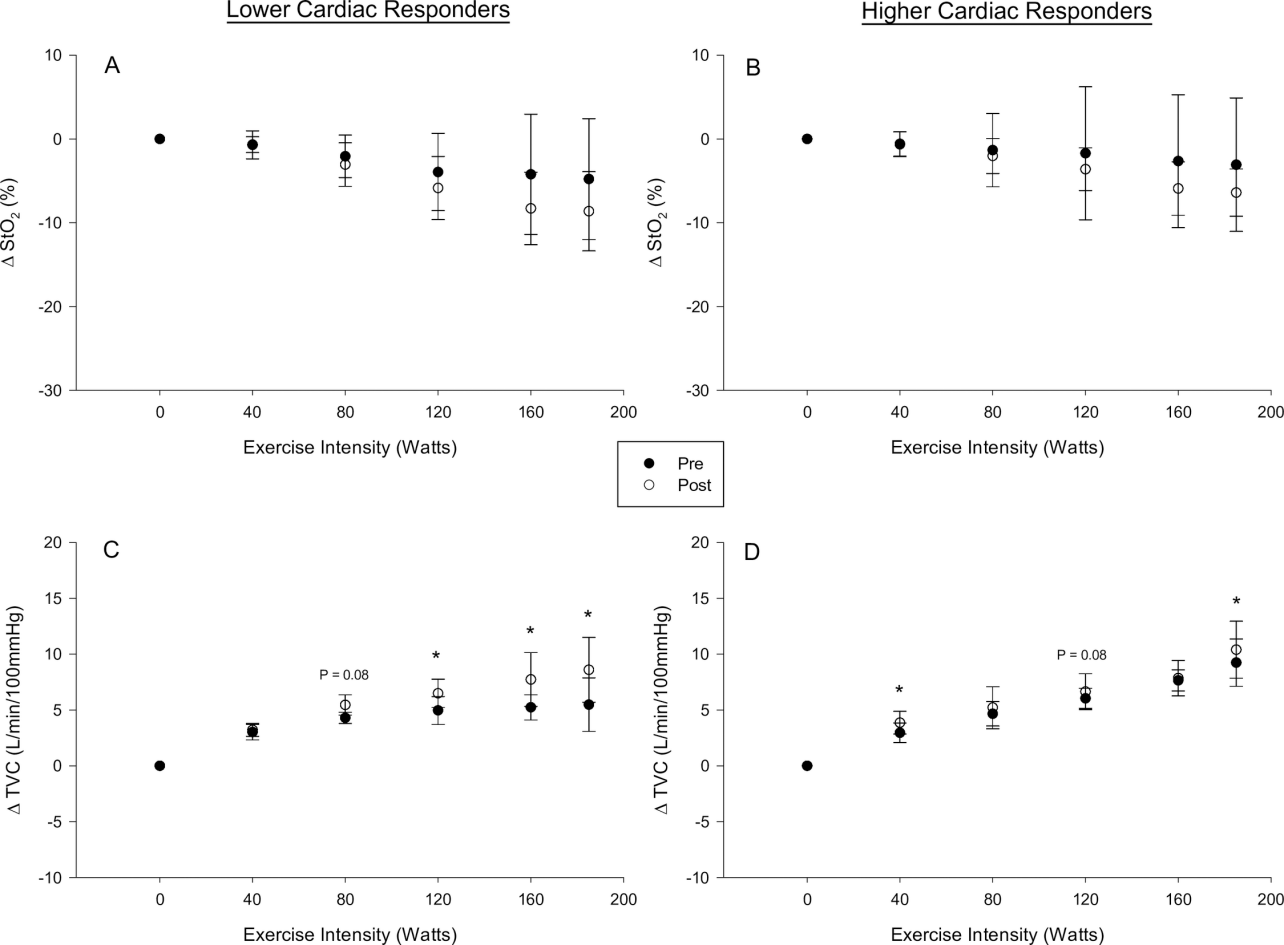
Exercise training and exercise skeletal muscle oxygenation. *Panel A-B*: Exercising skeletal muscle saturation. *Panel C-D*: Total vascular conductance. *Panel A and C*: Lower cardiac responders. *Panel B and D*: Higher cardiac responders. * denotes statistically significant difference between pre and post-training within a cardiac response group (P < 0.05).

Skeletal muscle saturation (%) was not different following exercise training in either lower or higher cardiac responders (F test P = 0.9). The increase in total vascular conductance (L/min/100mmHg) from baseline was elevated in lower cardiac responders at 120 W (P = 0.047), 160 W (P = 0.002) and 185 W (P = 0.001) while higher cardiac responders had an elevation at both 40 W (P = 0.009) and 185 W (P = 0.049) (Fig 4). The increase in total vascular conductance from baseline was reduced in lower cardiac responders compared to higher cardiac responders pre-training at 160 W (P = 0.004), 185 W (P = 0.042), but it was not different post-training (all P > 0.4). RPE_L_ (F test P = 0.7) or RPE_WB_ (F test P = 0.1) was unaffected by SIT.

## Discussion

In response to exercise training it is believed that $\dot{Q}$-$\dot{V}O_{2}$ is not affected [16, 18] as the submaximal cardiac response at a given $\dot{V}O_{2}$ does not change or is reduced [16–19, 21]. However, previous work failed to consider whether an individual’s pre-training $\dot{Q}$-$\dot{V}O_{2}$ determined whether SIT could achieve improvements in submaximal Q. Assessing exercise training effects and therefore the cardiovascular adaptations at the individual level remained poorly understood. Recently, we demonstrated considerable inter-individual variation in $\dot{Q}$-$\dot{V}O_{2}$ that was correlated with SV as opposed to C_a_O_2_ [11]. In the present study we tested the following hypotheses: 1) Lower cardiac responders would present with an elevated $\dot{Q}$-$\dot{V}O_{2}$ following SIT during subsequent submaximal exercise while higher cardiac responders would be unaffected. 2) An elevated submaximal $\dot{Q}$-$\dot{V}O_{2}$ would improve skeletal muscle oxygenation and reduce RPE. The primary novel findings of the study were as follows: 1) Lower cardiac responders had an elevated submaximal $\dot{Q}$-$\dot{V}O_{2}$ post-training while higher cardiac responders were unaffected by training. 2) An elevated $\dot{Q}$-$\dot{V}O_{2}$ in lower cardiac responders was due to an increase in SV, as HR was unchanged. 3) Skeletal muscle oxygenation and RPE were unaffected by the increased $\dot{Q}$-$\dot{V}O_{2}$.

These findings support the hypothesis that 4 weeks of SIT can increase $\dot{Q}$-$\dot{V}O_{2}$, providing an individual has a relatively low $\dot{Q}$-$\dot{V}O_{2}$ relationship prior to training. However these results do not support our secondary hypothesis that elevating $\dot{Q}$-$\dot{V}O_{2}$ would increase skeletal muscle oxygenation and reduce RPE in submaximal exercise.

### Exercise training and $\dot{Q}$-$\dot{V}O_{2}$

The conventional understanding of exercise training and $\dot{Q}$-$\dot{V}O_{2}$ arises from previous work from the late 1960s [16–18, 32], and mid 1980s [19] focusing on traditional endurance exercise training. These studies provided evidence for either no change [17–19] or a reduction [16, 32] in $\dot{Q}$ at the same $\dot{V}O_{2}$ or absolute exercise intensity post-training. Increases in SV that resulted from training were offset by a reduction in HR such that $\dot{Q}$ was unchanged [17–19].

SIT training in the present study was the Tabata protocol [24], a method of SIT in which participants complete supramaximal 20 second sprints on a cycle ergometer separated by a brief, 10 second active recovery period. SIT is a relatively new training method that produces similar improvements in cardiorespiratory fitness (peak capacity) compared to traditional endurance exercise, but in a fraction of the time [22, 23]. It is important to note that given the relatively recent emergence of SIT, the majority of literature on submaximal $\dot{Q}$ has focused on endurance exercise. There has been only one study to date that has investigated the effects of SIT training on submaximal $\dot{Q}$ [21] and this was in older, overweight, sedentary women. In that study, SIT training consisted of 4–7 sprints on a cycle ergometer at 5% body weight. Training sessions were completed 3 times per week for 4 weeks. Similar to the results from endurance training, the authors reported no change in submaximal $\dot{Q}$ following training at the pre-training relative intensity of 50% $\dot{V}O_{2}$ max. Importantly, studies evaluating the impact of exercise training on submaximal $\dot{Q}$-$\dot{V}O_{2}$ have all based their interpretation on group average responses without consideration for inter-individual differences in the pre-training $\dot{Q}$-$\dot{V}O_{2}$. Thus, it was previously unknown if SIT training can impact $\dot{Q}$-$\dot{V}O_{2}$ in young healthy males, but more importantly whether inter-individual differences in pre-training $\dot{Q}$-$\dot{V}O_{2}$ determine this response.

The surprising lack of research in this area despite prior observations of $\dot{Q}$ heterogeneity from the 1960s [12, 13] and 1980s [14] may stem from current dogma regarding the cause of $\dot{Q}$-$\dot{V}O_{2}$ heterogeneity. Namely, it is thought that $\dot{Q}$-$\dot{V}O_{2}$ differences between individuals are simply a reflection of differences in C_a_O_2_. This dogma is based on studies in which within-subject manipulation of blood oxygen content resulted in compensatory changes in $\dot{Q}$-$\dot{V}O_{2}$ as recently addressed by Adami *et al*. [10]. Therefore, any exercise training adaptations outside of changes influencing C_a_O_2_ are inconsequential. However, we recently demonstrated that inter-individual differences in $\dot{Q}$-$\dot{V}O_{2}$ are in fact strongly associated with SV but not C_a_O_2_ [11], suggesting to us that potential training adaptions influencing SV may differentially affect $\dot{Q}$-$\dot{V}O_{2}$ depending on initial $\dot{Q}$-$\dot{V}O_{2}$ of the individual.

Consistent with the exercise training literature and our hypothesis, $\dot{Q}$-$\dot{V}O_{2}$ in the higher relative cardiac responders was unaffected by exercise training. In contrast, lower cardiac responders had an elevated $\Delta \dot{Q}$ response post-training beginning at 120 W. The elevated $\Delta \dot{Q}$ was due to an increase in ΔSV, as ΔHR remained unchanged. Prior investigations assessing $\dot{Q}$-$\dot{V}O_{2}$ and exercise training may have missed this phenomenon by not identifying disparate cardiac response groups *a priori*, such that a potential training effect was masked when averaged over all participants. In this context, our findings are important because they identify for the first time that submaximal exercise $\dot{Q}$-$\dot{V}O_{2}$ actually can be improved with training in young healthy males provided they initially have a low $\dot{Q}$-$\dot{V}O_{2}$ response. These findings speak to the importance of considering inter-individual differences when assessing the efficacy of training studies or designing training protocols. They also raise a new set of questions regarding what the underlying mechanisms are that can explain why such improvement occurs only in lower $\dot{Q}$-$\dot{V}O_{2}$ responders.

In the lower cardiac response group, morphological and functional adaptions in response to SIT could be facilitating the observed increase in ΔSV. Echocardiographic assessments following a combination of endurance/SIT and high intensity interval runs demonstrated increases in left ventricular end diastolic volume and mass that resulted in an increased ejection fraction and SV [33]. This observation was confirmed with cardiovascular magnetic resonance imaging following high intensity interval training [34]. Additionally, mice models have demonstrated that aerobic interval training improves both cardiomyocyte contractility and calcium handling [35], potentially providing another mechanism of improved ΔSV post-training. Therefore the elevated $\Delta \dot{Q}$ response in lower cardiac responders could reflect greater changes in left ventricular mass and cardiomyocyte functionality, although these outcomes were not measured and remain to be determined.

### Exercising muscle oxygenation, RPE and blood flow distribution

Exercising skeletal muscle oxygenation results from the complex interaction of skeletal muscle blood flow and arterial blood pressure regulation. Increases in skeletal muscle blood flow arise principally due to an increase in local vascular conductance [36], which may be restrained by the presence of sympathetic vasoconstriction as part of arterial blood pressure regulation [36]. Of course the increased skeletal muscle blood flow must be matched by the increase in $\dot{Q}$ or require the addition of vasoconstriction in non-exercising tissue to add some flow redistribution if the increase in $\dot{Q}$ is inadequate [37, 38].

A potential benefit of an increase in the $\dot{Q}$ response in submaximal exercise would be that the regulation of arterial blood pressure could be achieved at a higher exercising muscle vascular conductance, with subsequently greater exercising skeletal muscle blood flow [37]. Based on this, we hypothesized that an increase in submaximal $\Delta \dot{Q}$ with exercise training would result in an increase in exercising muscle oxygenation via an increase in blood flow. However, contrary to our hypothesis, an elevated submaximal $\Delta \dot{Q}$ was not associated with improved skeletal muscle oxygenation as saturation was not different following exercise training in lower cardiac responders. Furthermore, skeletal muscle saturation was not different between cardiac response groups before or after training.

Exercising muscle saturation via NIRS is used as a surrogate measure of the balance in exercising muscle between oxygen delivery via perfusion and oxygen consumption [39, 40]. Given that there was no difference in the change in skeletal muscle saturation between higher and lower $\dot{Q}$ responders prior to exercise training it would appear that the lower cardiac responder’s integration of central cardiac and peripheral vascular bed responses allowed the same exercising muscle oxygen delivery via redistribution of some perfusion from non-exercising tissue. We hypothesized that improved $\dot{Q}$-$\dot{V}O_{2}$ following training may allow for improved exercising muscle perfusion at the same $\dot{V}O_{2}$, and therefore improved oxygenation. However, we did not observe such an improvement. Rather, it appears that when submaximal $\Delta \dot{Q}$ was elevated following SIT, this increase in systemic blood flow was linked with a reduction in perfusion redistribution, as the exercising muscles already had normal perfusion. In other words, less sympathetically mediated vasoconstriction of non-exercising tissue following exercise training in lower cardiac responders. Indeed, lower cardiac responders had an increase total vascular conductance from 120 W to 185 W thus lending support to this inference. This intriguing possibility awaits additional invasive studies to more comprehensively assess the interaction of systemic blood flow and regional distribution of that blood flow.

Given that the effect of an increase in $\Delta \dot{Q}$ was associated with an increase in total vascular conductance such that saturation was not different, it is perhaps not surprising that both RPE_WB_ and RPE_L_ were not different following training in lower cardiac responders. Hyperoxic, hypoxic and skeletal muscle perfusion reductions have demonstrated the sensitivity of RPE to oxygen delivery during exercise [6, 7, 41]. In the absence of a measureable change in skeletal muscle saturation, the present RPE observations align with our understanding oxygen delivery effects on RPE.

### Experimental considerations

Claims of an altered $\dot{Q}$-$\dot{V}O_{2}$ relationship are predicated on accurate measures of $\dot{Q}$ and $\dot{V}O_{2}$. When exercising at an absolute exercise intensity, in the absence of any changes in metabolic efficiency, $\dot{V}O_{2}$ should be similar. Indeed in the present study we observed superimposable exercise intensity—$\dot{V}O_{2}$ relationships before and after training (F test P = 0.4). We are therefore confident in our measures of $\dot{V}O_{2}$.

$\dot{Q}$ was measured using Innocor up to 160 W. With this method there is the potential for an underestimation of $\dot{Q}$ due to soluble gas recirculation and the associated effects on the concentration gradient required for diffusion of the soluble gas from the rebreathing bag into the pulmonary circulation. Potential issues have been addressed previously [11] with the identification that, based on the measured $\dot{Q}$, mixed venous C_v_O_2_ was well within plausible values (C_v_O_2_ ≥ 20 ml O_2_/L). In the current study, there was no difference in the duration of the rebreathe maneuver before and after training within a cardiac or between cardiac response groups (F test P = 0.5). Breathing maneuver times ranged from 20 ± 3 seconds at rest ($\dot{V}O_{2}$ of 0.4 ± 0.1 L/min) to 13 ± 1 seconds at 160 W ($\dot{V}O_{2}$ of 2.3 ± 0.1 L/min). We also coached each individual through his unique breathing pattern (both tidal volume and breathing frequency) as to not elicit hyperventilation, which can influence $\dot{Q}$ [42, 43]. Beyond 160 W, $\dot{Q}$ implementation of the Finometer correction was equally effective as correction variance was not different before and after training within a cardiac or between cardiac response groups (F test P = 0.2). Therefore, it is highly unlikely that recirculation or the application of Innocor-corrected Finometer $\dot{Q}$ measures above 160 W (i.e. measurement error) explain the differences in $\dot{Q}$-$\dot{V}O_{2}$ between lower and higher cardiac responders.

Lastly, we computed systemic C_v_O_2_ to assess $\dot{Q}$ validity. In doing so, we were able to establish the mixed venous C_v_O_2_ given measured C_a_O_2_, $\dot{V}O_{2}$ and $\dot{Q}$. From our 264 measures of $\dot{Q}$, all were plausible (C_v_O_2_ ≥ 20 ml O_2_/L). One participant was identified as having an implausible C_v_O_2_ (0 < C_v_O_2_ < 20 ml O_2_/L) at 160 W pre-training and was removed from all data analysis. We are therefore confident that: 1) individuals differ in their $\dot{Q}$-$\dot{V}O_{2}$ relationship and can be classified as lower and higher cardiac responders and 2) that SIT increases the submaximal $\dot{Q}$-$\dot{V}O_{2}$ relationship in lower cardiac responders while it has no effect in higher cardiac responders.

## Conclusion

In conclusion, we provide the first evidence that an individual’s $\dot{Q}$-$\dot{V}O_{2}$ determines whether they experience an increase in submaximal exercise $\dot{Q}$. Specifically, only lower cardiac responders are sensitive to SIT and present with an elevated $\dot{Q}$-$\dot{V}O_{2}$ post-training. The increase in $\dot{Q}$-$\dot{V}O_{2}$ post-training was mediated by an increase in SV with no change in HR. These findings emphasize the importance of recognizing individual response differences in attempting to understand the integrated cardiovascular response to exercise and to exercise training. It also indicates the potential importance of considering such differences in selecting efficacious training modalities.

Interestingly, increased $\dot{Q}$-$\dot{V}O_{2}$ in lower responders following SIT did not improve skeletal muscle oxygenation and RPE. This finding suggests a reduced sympathetic vasoconstriction in non-exercising tissue following SIT in lower cardiac responders. This hypothesis remains to be tested with follow-up exercise imaging and invasive studies.

## Supporting information

S1 File PLoS ONE raw data submission FINAL. Raw data file for the present study.
(XLSX)
